# Complete mitochondrial genome and the phylogenetic position of the pelagic octopus *Tremoctopus violaceus* (Mollusca: Tremoctopodidae)

**DOI:** 10.1080/23802359.2018.1532347

**Published:** 2018-10-26

**Authors:** Yuh-Wen Chiu, Chih-Wei Chang, Kang-Ning Shen, Yu-Min Ju, Hung-Du Lin

**Affiliations:** a Center for Research in Water Science and Technology, National Cheng Kung University, Tainan, Taiwan;; b Department of Hydraulic and Ocean Engineering, National Cheng Kung University, Tainan, Taiwan;; c National Museum of Marine Biology and Aquarium, Pingtung, Taiwan;; d Department of Marine Biology, Graduate Institute of Marine Biology, National Dong Hwa University, Pingtung, Taiwan;; e Aquatic Technology Laboratories, Agricultural Technology Research Institute, Hsinchu, Taiwan;; f Department of Biology, The Affiliated School of National Tainan First Senior High School, Tainan, Taiwan

**Keywords:** Argonautoidea, mitogenome, octopods, *Tremoctopus violaceus*

## Abstract

*Tremoctopus violaceus* is a small species of pelagic octopods which inhabit in subtropical and tropical open oceans. The mitogenome of *T. violaceus* is 16,015 base pairs (bp) in length and contained 13 protein-coding genes, two rRNA genes, and 22 tRNA genes. Sequence analysis showed that the overall base composition is 31.5% for A, 40.47% for T, 7.79% for C, and 20.24% for G. The newly characterized complete mitochondrial genome of *T. violaceus* will provide essential data for further studies of this endangered species.

The superfamily Argonautoidea Naef, 1912 (Class Cephalopoda, Phylum Mollusca) is also characterized by marked sexual dimorphism with males small or dwarf (Naef 1923) and comprises four monogeneric families: Argonautidae, Tremoctopodidae, Ocythoidae and Alloposidae. These species are characterized by an unusual mating system which involves the males detach a modified third arm, or hectocotylus (the modified arm in males used for transferring spermatophores to the female) from the male to the mantle cavities of the females. The family Tremoctopodidae contains the single genus *Tremoctopus* (blanket octopus) of which there are four known species (Mangold et al. 2008).

*Tremoctopus violaceus* Delle Chiaje, 1830 belongs to the family Tremoctopodidae, is a small species of pelagic octopods which inhabit in subtropical and tropical open oceans (Mangold et al. 2008). Previous studies suggested that a sister taxon relationship was recovered between Argonauta and Ocythoe and between Tremoctopus and Haliphron using sequences of 12S rDNA, 16S rDNA, COI and COIII (Strugnell and Allcock 2010). Systematic studies of Argonautoidea that lead to elucidate molecular evolutionary relationships and to facilitate understanding of the evolution of morphological characters within the group. In this investigation, the complete mitochondrial genome of *T. violaceus* was sequenced and characterized in Taiwan using next-generation sequencing technology, which provides more molecular data for phylogenetic analysis of this species (Figure 1).

**Figure 1.**
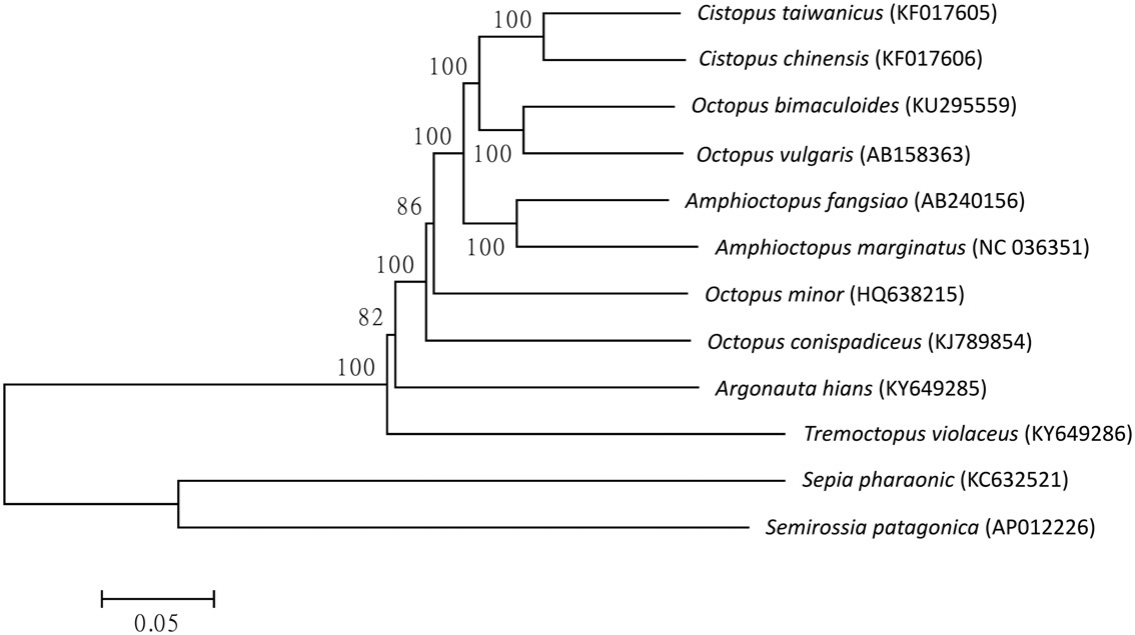
Phylogenetic relationship of *Tremoctopus violaceus* with other Octopoda as inferred by entire mitogenome. Trees were reconstructed using MEGA 7 program (Kumar et al. 2016) with neighbour-joining method. Numbers above branches are bootstrap values by 1000 replicates. The phylogenetic tree showed that a basal position of *T. violaceus* in Octopoda.

Specimen of *T. violaceus* was obtained from Taiwan and deposited at the National Museum of Marine Biology and Aquarium, Pingtung, Taiwan. Genomic DNA extraction and next-generation sequencing were described in previous publication (Chiu et al. 2017). Initially, the raw next-generation sequencing reads generated from HiSeq 2000 (Illumina, San Diego, CA) were altered to remove low-quality reads. Around 0.06% raw reads (7944 out of 13,849,558) were subjected to de novo assembly using commercial software (Geneious V9, Auckland, New Zealand) to produce a single, circular form of complete mitogenome with about an average 122× coverage. The protein coding, rRNA and tRNA genes of *T. violaceus* mitogenome were predicted by using MITOS (Bernt et al. 2013) tool and manually inspected. The complete mitogenome of *T. violaceus* consists of 13 protein-coding genes (PCGs), 22 transfer RNAs (tRNAs), two ribosomal RNAs, and one putative control region. The complete mitogenome of *T. violaceus* is a circular double-stranded DNA sequence that is 16,015 bp long (GenBank accession no. KY649286) and was biased toward A + T content at 71.97% (31.5% A, 40.47% T, 7.79% C, and 20.24% G). Eight of the 13 PCGs (NAD6, NAD4L, NAD3, COB, COX1, COX2, ATP8, and COX3) used ATG as start codon, NAD1 and NAD2 used ATA, NAD4, and NAD5 used ATA and another one (ATP6) used ATT. According to the tree topology, *T. violaceus* is close to *Argonauta hians* and both of them are in the same superfamily of Argonautoidea. The phylogenetic relationship suggested a basal position of *T. violaceus* in Octopoda. In conclusion, the complete mitogenome of the *T. violaceus* deduced in this study provides essential and important DNA molecular data for further phylogenetic and evolutionary analysis for Argonautoidea.

## References

[CIT0001] Bernt M, Donath A, Jühling F, Externbrink F, Florentz C, Fritzsch G, Pütz J, Middendorf M, Stadler PF 2013 MITOS: improved de novo metazoan mitochondrial genome annotation. Mol Phylogenet Evol. 69:313–319. 22982435 10.1016/j.ympev.2012.08.023

[CIT0002] Chiu YW, Chang CW, Lin HD, Shen KN 2017 The complete mitogenome of the winged argonaut *Argonauta hians* and its phylogenetic relationships in Octopoda. Conservation Genet Resour. 10:359–362.

[CIT0003] Kumar S, Stecher G, Tamura K 2016 MEGA7: molecular evolutionary genetics analysis version 7.0 for bigger datasets. Mol Biol Evol. 33:1870–1874. 27004904 10.1093/molbev/msw054 PMC8210823

[CIT0004] Mangold KM, Vecchione M, Young RE 2008 Tremoctopodidae Tryon, 1879. Tremoctopus Chiaie. 1830: Blanket octopus. Version 16 October 2008. [Accessed 2014 November 13]. Available from: http://tolweb.org/Tremoctopus/20202/2008.10.16 in The Tree of Life Web Project, http://tolweb.org/.

[CIT0005] Naef A 1923 Cephalopoda. Fauna e Flora del Golfo di Napoli (Translated from German by the Israel Program for Scientific Translations, Jerusalem, 1972). Monograph. 35:1–917.

[CIT0006] Strugnell J, Allcock AL 2010 Co-estimation of phylogeny and divergence times of Argonautoidea using relaxed phylogenetics. Mol Phylogenet Evol. 54:701–708. 19941965 10.1016/j.ympev.2009.11.017

